# Seasonal fluxes of carbon monoxide from an intensively grazed grassland in Scotland

**DOI:** 10.1016/j.atmosenv.2018.09.039

**Published:** 2018-12

**Authors:** N. Cowan, C. Helfter, B. Langford, M. Coyle, P. Levy, J. Moxley, I. Simmons, S. Leeson, E. Nemitz, U. Skiba

**Affiliations:** Centre for Ecology and Hydrology, Bush Estate, Penicuik, EH26 0QB, UK

**Keywords:** Eddy covariance, Meteorology, Greenhouse gas, Inventory, CO

## Abstract

Fluxes of carbon monoxide (CO) were measured using a fast-response quantum cascade laser absorption spectrometer and the eddy covariance method at a long-term intensively grazed grassland in southern Scotland. Measurements lasted 20 months from April 2016 to November 2017, during which normal agricultural activities continued. Observed fluxes followed a regular diurnal cycle, peaking at midday and returning to values near zero during the night, with occasional uptake observed. CO fluxes correlated well with the meteorological variables of solar radiation, soil temperature and soil moisture content. Using a general additive model (GAM) we were able to gap fill CO fluxes and estimate annual fluxes of 0.38 ± 0.046 and 0.35 ± 0.045 g C m^−2^ y^−1^g C m^−2^ y^−1^ for 2016 and 2017, respectively. If the CO fluxes reported in this study are representative of UK grasslands, then national annual emissions could be expected to be in the order of 61.91 (54.3–69.5) Gg, which equates to 3.8% (3.4–4.3%) of the current national inventory total.

## Highlights

• A long term dataset of CO fluxes measured from agricultural grasslands.
• A potentially large source of CO emissions is quantified from UK grasslands.
• CO emissions from grassland can be modelled using meteorological measurements.

## 1 Introduction

Carbon monoxide (CO) is considered both a primary and secondary atmospheric pollutant due to its direct repercussions on human health and its participation in atmospheric chemistry. The direct health impacts of elevated CO concentrations (i.e. > 1000 ppm) are typically associated with time spent in close proximity to the burning of fossil fuels and biomass (i.e. urban areas and stove cooking with poor ventilation) (Townsend, 2002; Raub et al., 2000). At regional scales, atmospheric concentrations of CO are generally well below those considered to be harmful to people (i.e. < 0.3 ppm).

The role that CO plays in atmospheric chemical processes involving trace gases remains important despite its relatively low concentrations. CO is the largest global sink of hydroxyl radicals (OH) in the atmosphere. OH radicals are a catalyst in the reaction which produces a net increase in tropospheric ozone (O_3_) in the presence of nitrogen oxide (NO_x_) compounds. Tropospheric O_3_ is known to be a strong greenhouse gas as well as being associated with respiratory problems and decreasing crop yields (Ainsworth et al., 2012). Conversely, destruction of OH reduces the atmospheric capacity to oxidise the important greenhouse gas methane (CH_4_), thereby indirectly increasing its lifetime and global warming potential (GWP) (Daniel and Solomon, 1998). With an estimated 120 Pg of carbon in the form of CO released from terrestrial systems (natural and anthropogenic) per year (IPCC, 2013), understanding potential sources and sinks of this influential compound at regional scales is important when evaluating the full impact of other significant air pollutants such as O_3_, NO_x_ and CH_4_.

The largest global sources of atmospheric CO are the incomplete combustion of fossil fuels and biomass, primarily due to anthropogenic activities with a contribution from naturally occurring events such as forest fires (Duncan et al., 2007). Photo-degradation (including photo-oxidation) and thermal degradation in both terrestrial and aquatic ecosystems as well as organic carbon in the atmosphere (i.e. oxidation of CH_4_) are also considered significant sources of global CO emissions (Khalil and Rasmussen, 1990; Weston, 2001). Photo-degradation is the breakdown of organic matter by radiation, a predominantly natural process which occurs during daylight hours when UV and visible radiation are present (Valentine and Zepp, 1993; L. A. Brandt et al., 2010). The absolute endpoint of photo-oxidation is the complete breaking down of carboxyl bonds to form CO_2_, but this process also generates CO (Miller and Zepp, 1995). Photo-degradation can occasionally be considered an important source of CO from soils and aquatic systems that are rich in organic materials, especially in arid ecosystems which have very low microbial activity. In such environments, abiotic processes account for the vast majority of naturally occurring carbon exchange with the atmosphere (King et al., 2012).

Under aerobic conditions, thermal degradation of carbon compounds can occur at relatively low temperatures (<100 °C), resulting in emission of trace gases such as CO_2_, CH_4_ and CO. This process is likely to occur in warm, dry soils, and accelerates with increasing temperature (Conrad and Seiler, 1985; Asperen et al., 2015). There is some evidence that rewetting soils after exposure to drought and high temperatures can generate a spike in CO production as water reacts with molecules generated by thermal degradation (Moxley and Smith, 1998). Studies have shown that the thermal degradation of organic materials may have a particularly significant impact on CO emissions from warm carbon rich ecosystems (Lee et al., 2011; Asperen et al., 2015).

Thermal degradation is also considered the primary source of CO emission from composting of organic materials and animal waste (Hellebrand. 2001). However, precise knowledge of chemical pathways influenced by thermal degradation in natural systems remains elusive due to the numerous interacting processes and conditions observed in soils and composting organic materials, such as microbial activity and the heterogeneous availability of oxygen.

Microbial activity in soils is generally considered a net CO sink, wherein it is consumed by oxidation processes and converted into CO_2_ (King, 2000; Pihlatie et al., 2016; Yonemura et al., 2000). Although microbial uptake of CO has long been proven to occur (Inman et al., 1971), the exact chemical pathways of these processes in natural soils remain difficult to quantify due to the large variety of competing organisms such as bacteria and fungi capable of CO oxidation in soils (Conrad and Seiler, 1985). It has been estimated that microbial uptake of CO in soils is approximately four times greater than emission at a global scale, with much of the microbial activity occurring in high carbon tropical regions around the equator where temperatures are consistently high (>30 °C) and damp enough to support thriving microbial communities (Liu et al., 2018).

Other sinks of CO include chemical reactions in the atmosphere, primarily the reaction with OH radicals in the troposphere (Jaffe, 1968). OH radicals are the result of excited oxygen atoms (generated via the photolysis of ozone or NO_x_) reacting with water vapor. OH radicals react with CH_4_ and CO molecules thereby removing CO from the atmosphere. It can therefore be stated that CO concentrations will decrease in the presence of sunlight when O_3_ and NO_x_ is available; however, the reaction is not immediate and the lifetime of atmospheric CO is generally in the region of two months (Khalil and Rasmussen, 1990).

Considering the impact that CO has in regards to potential global radiative forcing caused by O_3_ and CH_4_, as well as the damaging effect that elevated O_3_ concentrations may have on plant health (Avnery et al., 2011), understanding CO emissions from agricultural areas is important in determining potential environmental damage caused by land management practices. Long term studies of atmospheric CO exchange with agricultural soils are still relatively rare with only a handful of experiments reported in literature, primarily carried out in the northern hemisphere. These studies provide a varied account of the surface-atmosphere interactions of CO and are by no means conclusive when addressing regional and global scale accounting. In this study we aim to quantify annual fluxes of CO from an intensively grazed grassland and identify meteorological drivers that may control these emissions.

## 2 Methodology

### 2.1 Field site management & meteorology

Measurements were made between April 2016 and November 2017 over an intensively grazed grassland field site at the Easter Bush Estate (Midlothian, Scotland) (Jones et al., 2017). The soil is a clay loam with a sand/silt/clay texture of 26/20/55 in the top 30 cm with a pH of 5.1 (in H_2_O). The soil is classed as imperfectly drained Macmerry soil of the Rowanhill association (eutric cambisol, FAO classification). Instrumentation was set up inside a permanently stationed, temperature controlled cabin with access to mains power. The cabin was located directly between two similarly managed grazed grasslands (each approximately 5.4 ha). A small road ran along the south fence line of the most southern field which saw an increase in traffic during rush hour times (morning between 6 and 9 a.m. and evening between 4 p.m. and 8 p.m.). The grassland fields had been predominantly used as high intensity grazing pasture for sheep (0.7 LSU ha^−1^) for over twenty years before measurements took place. Their management is typical for this region, with predominately ammonium nitrate fertilization (but urea in the year of this study), in spring, early and mid-summer; liming every 3–5 years to maintain the pH between 5.5 and 6 and occasional ploughing and reseeding. In May 2016, the sheep were removed from the South Field and the grass was grown as silage crop, harvested in July (approximately 50 cm grass height). Sheep were returned to the field in mid-August. In 2017 and at all other times, both fields were managed identically with sheep grazing throughout the year at 0.7 LSU ha^−1^ (approximately 7 cm grass height). Urea fertilizer (70 kg ha^−1^) was applied to both fields on the same days and in similar quantities during the growing seasons (2016: 13/06, 26/07, 23/08 and 2017: 21/05, 22/06, 04/08).

**Fig. 1:**
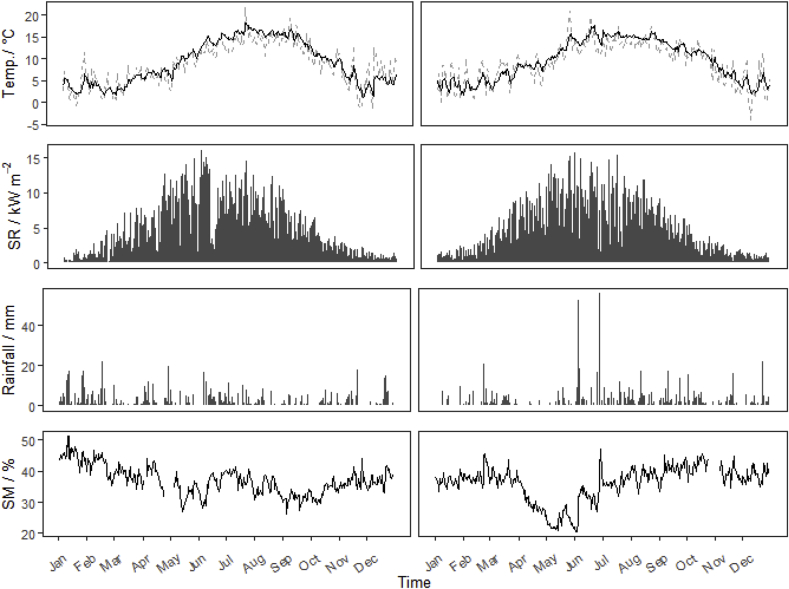
Measured soil temperature (black), air temperature (grey)(top), solar radiation (SR), rainfall (bar) and volumetric soil moisture content (SM) for the years 2016 (left) and 2017 (right).

Measurements of soil temperature (0.35 m depth), air temperature (1.8 m height) and rainfall (tipping bucket) were made at the field site throughout the measurement period and averaged every 30 min (Fig. 1). Soil moisture was also measured by a cosmic-ray moisture sensor (Hyroinnova CRS-2000) (Köhli et al., 2015) at the COSMOS-UK Easter Bush measurement site (www.cosmos.ceh.ac.uk), located approximately 300 m north of the flux mast. Meteorological observations over the two years were not markedly different, with cumulative annual rainfall of 778 and 770 mm for 2016 and 2017, respectively. Solar radiation measured at the site (Pyranometer, SKS1110, Skye Instruments, Powys, UK), showed a consistent trend across both years of measurements.

### 2.2 Eddy covariance measurements

**Fig. 2:**
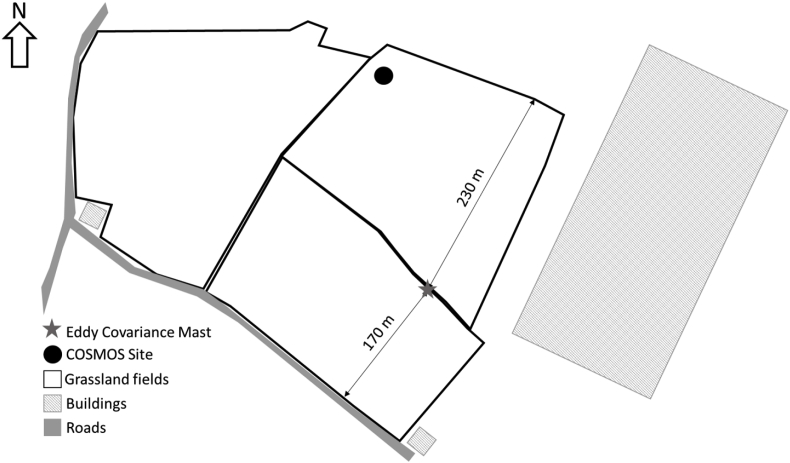
The eddy covariance mast and meteorological measurement equipment were positioned in a small enclosed area between two grazed grassland fields at the Easter Bush permanent field site, Midlothian, Scotland.Fluxes were calculated at 30 min intervals using the EddyPro software (Version 6.2.1, Li-COR, Lincoln, NE, U.S.A.), based on the covariance between gas concentration (χ) and vertical wind speed (w) (Equation (1)). For flux data taken with a low signal-to-noise ratio, timelag identification by maximisation of the cross covariance between concentration (χ) and vertical wind component (*w*) introduces systematic biases (Langford et al., 2015). Instead, the timelag was estimated on a daily basis for CO, taking the maximisation of covariance of data over a 24 h period. This timelag was then fixed for all data within the nominal 24-h chunk and fluxes were calculated on a 30 min basis.

An eddy covariance mast was erected several meters from the cabin with an ultra-sonic anemometer (WindMaster Pro 3-axis, Gill, Lymington, UK) mounted at 2.5 m to measure fluctuations in 3-D wind components at a frequency of 20 Hz. The prevailing wind is from the southwest, providing measurements from the South Field (Fig. 2). A 14 m length of 1/4” ID Decabon tubing was attached to the mast near the sonic anemometer (northward, eastward and vertical separation from the center of the sonic of 14, 4 and 15 cm, respectively). This inlet was run back along a protected conduit into the temperature controlled cabin where it was connected to a continuous wave quantum cascade laser (QCL) absorption spectrometer gas analyser (CW-QC-TILDAS-76-CS, Aerodyne Research Inc., Billerica, MA, USA) which had been fitted with a laser capable of measuring atmospheric CO at 10 Hz with instrumental noise less than 0.1 ppb, together with N_2_O and H_2_O, using an absorption feature at 22.0 m^-1^. The TDLWintel software (Aerodyne Research Inc., Billerica, MA, USA), fits the observed spectra to a template of known spectral line profiles from the HITRAN (HIgh-resolution TRANsmission) molecular spectroscopic database. Absolute trace gas concentrations can then be calculated from the strength of the absorption line measured, the temperature and pressure of the absorption cell and the path length with an absolute signal for concentration data accurate to within 3%. A vacuum pump (Triscroll 600, Agilent Technologies, US) was used to draw air through the inlet and instrument with a flow rate of approximately 14 l min^−1^. Data from the sonic anemometer and QCL was logged in tandem using a custom program written in LabView™ (National Instruments, TX, USA).

[1] $$F\chi =\bar{\chi 'w'}$$

In the flux calculation processing, we applied double coordinate rotation (vertical and crosswind), spike removal, block averaging and outlier removal of artefact measurements. Correction for the frequency response of the system, both high and low-frequency losses, were made using the analytical method of Moncrieff et al. (1997). Corrections for density fluctuations due to temperature fluctuations were applied on a half-hourly basis using the method of Ibrom et al. (2007). The quality control scheme of Foken (2003) was used to remove poor quality flux measurements (category 5 or above). Data were also rejected on the basis of extreme outliers and friction velocity (u_*_) values less than 0.1 m s^−1^. Only data in which at least 90% of the flux came from a radius of 150 m from the flux tower and data in which the peak contribution to the flux was at least 25 m from the tower were used in this study, based on the calculations of Kljun et al. (2004). Flux random uncertainty was estimated by the method of Finkelstein and Sims (2001) integrated over a fixed 10 s correlation period. This was chosen because the estimation methods of the integral time scale of the turbulence become uncertain for noisy data.

### 2.3 Concentrations of NO and NO_2_

Concentrations of NO and NO_2_ were measured at the station by a chemiluminescence NO-NO_2_-NO_x_ analyser (Thermo 42C, Thermo Electron Corp., MA, USA). The inlet was positioned on a separate mast at 1.8 m. Measurements were logged every 10 s, then averaged to give 30 min mean values. The NO and NO_2_ (NO_x_) measurements are part of a long term monitoring network, but were useful in this experiment as an indicator of fossil fuel combustion. It is known that CO, NO and NO_2_ are all by-products of fuel combustion in vehicles, therefore NO_x_ may indicate if fossil fuel emissions in the local area are having an impact on measured CO fluxes.

### 2.4 Gap filling

A general additive model (GAM) was fitted to the measured CO fluxes in order to parametise them based on meteorological observations. This approach accounts for nonlinear responses to environmental variables by fitting a smooth response with cubic splines, as implemented using the mgcv package in the R software (Wood, 2006). Only CO flux data that passed quality control was included in the model fit which explicitly did not predict any temporal variation in the measurements. The meteorological variables included in the CO flux fit were solar radiation, soil temperature (0.35 m) and soil moisture content (approximately 0–50 cm). The degree of smoothing was optimised by the algorithm, with all points given equal weighting. Predictions from the GAM in this study were used to compare whether fluxes of CO could be attributed to changes in meteorological conditions or if there were other factors influencing the emissions.

## 3 Results

### 3.1 CO concentration and fluxes

Atmospheric concentrations of CO at the field site varied over the measurement period with monthly averages varying from 0.105 to 0.187 ppm (Table 1). The general trend was for CO concentrations to increase during the colder months of winter, likely due to an increased burning of fossil fuels in the wider surrounding area.

**Table 1:** A summary of CO concentrations and fluxes measured at the Easter Bush field site from April 2016 to November 2017. The mean, standard deviation and percentiles of measurement data are calculated on a monthly basis.

Date		CO Mixing Ratio (ppm)			CO Flux (nmol m^−2^ s^−1^)		
Year	Month	Mean	St.dev	Percentiles (25–75%)	Mean	St.dev	Percentiles (25–75%)
2016	April	0.160	0.008	0.156–0.163	0.85	1.47	−0.33–1.72
2016	May	0.133	0.005	0.129–0.137	0.89	1.38	−0.35–1.78
2016	June	0.118	0.011	0.108–0.127	1.41	1.46	0.61–2.15
2016	July	0.105	0.007	0.102–0.109	1.86	1.73	0.66–2.65
2016	August	0.113	0.012	0.103–0.121	1.52	1.67	0.42–2.51
2016	September	0.112	0.006	0.109–0.115	0.45	1.28	−0.47–1.26
2016	October	0.139	0.023	0.126–0.147	1.02	1.55	−0.08–1.77
2016	November	0.144	0.009	0.137–0.151	0.8	1.17	0.14–1.43
2016	December	0.143	0.014	0.133–0.147	1.07	1.29	0.26–1.93
2017	January	0.187	0.019	0.169–0.203	0.88	1.43	0.32–1.82
2017	February	0.166	0.024	0.152–0.174	1.04	1.63	−0.17–2.11
2017	March	0.157	0.011	0.151–0.162	1.38	1.57	0.29–2.36
2017	April	0.153	0.008	0.151–0.156	0.32	1.5	−0.83–1.31
2017	May	0.141	0.017	0.129–0.151	0.16	1.63	−1.08–1.32
2017	June	0.117	0.008	0.112–0.121	1.27	1.64	0.25–2.2
2017	July	0.110	0.006	0.106–0.114	1.83	1.59	0.58–2.86
2017	August	0.123	0.012	0.115–0.127	1.74	1.48	0.54–2.52
2017	September	0.151	0.013	0.145–0.153	1.35	1.32	0.44–2.13
2017	October	0.150	0.011	0.145–0.154	0.77	1.26	−0.18–1.48
2017	November	0.154	0.007	0.149–0.158	0.94	1.33	0.24–1.56

**Fig. 3:**
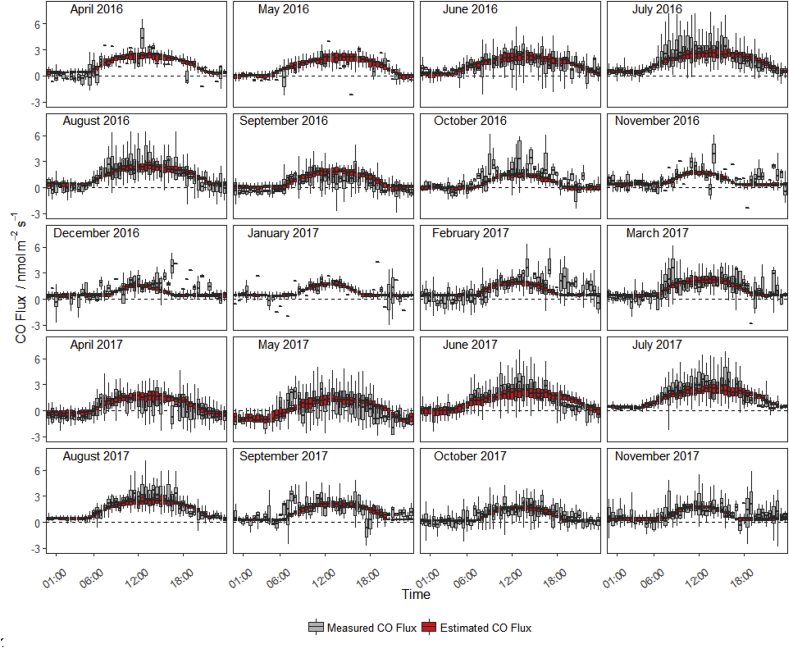
Diurnal trends in the measured CO flux and the CO flux estimated based on the GAM fit with meteorological variables. Box plots represent the median, quartiles and 95% confidence intervals of data binned on an hourly basis for each month.

A diurnal trend was observed in fluxes of CO throughout the 20 month measurement period with fluxes peaking during the day (approximately 1–4 nmol m^−2^ s^−1^) then falling to values near zero or negative (typically no further than −1 nmol m^−2^ s^−1^) during the night (Fig. 3). The highest daytime peaks occurred in the warmest months of summer (June, July and August). A smaller fraction of the measured fluxes passed quality control criteria during the winter (December, January, February) due to unfavourable weather conditions (i.e. low winds and stable stratification); however, diurnal trends are still identifiable in the remaining data.

Fitting a GAM between the measured CO fluxes and meteorological variables provide a simple means by which to determine the influence of these variables on CO fluxes from the field. The direct comparison of the measured flux and the flux predicted by the GAM at a 30 min interval (R^2^ = 0.37) is reasonable considering the relatively large random uncertainty of the measured fluxes (mean random absolute error 0.31 nmol m^−2^ s^−1^ and median relative error of 0.39). A total of 66% of the GAM predicted flux values fall within the 95% confidence interval of the real flux measurements. The GAM prediction does well when describing the diurnal trend in measurements as it varies between the separate months (Fig. 3). The largest divergence from the GAM fit typically occurs during the winter months (December, January and February), especially during the morning (between 6 and 9 a.m.) and evening (between 4 p.m. and 8 p.m.). These times correlate with when we would expect the highest levels of traffic and fuel burning to occur in the local surroundings, suggesting that some contamination may be affecting the measured fluxes.

### 3.2 Investigating contamination from fossil fuel combustion

**Fig. 4:**
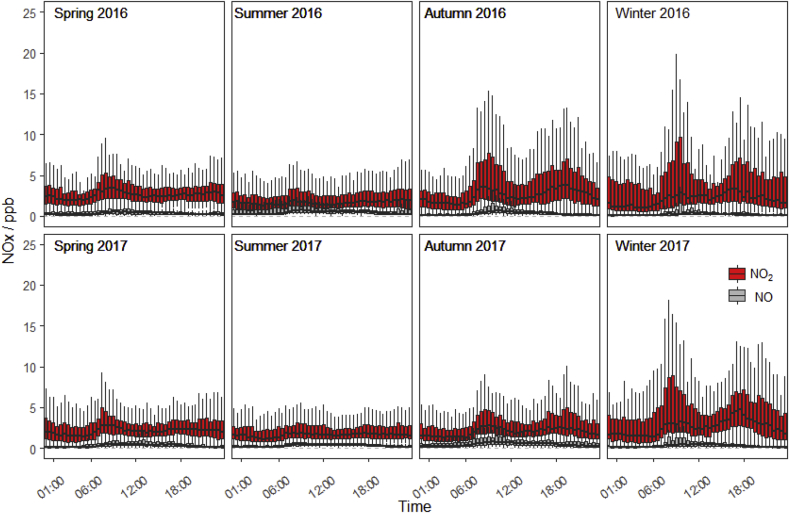
Concentrations of NO and NO_2_ measured at the site. Box plots represent the median, quartiles and 95% confidence intervals of data binned on an hourly basis for each month.

Measurements at the site reveal that concentrations of NO and NO_2_ increased markedly during the colder months of the year (Fig. 4). These emissions are likely to have originated from surrounding combustion sources, primarily cars exhausts and heating systems within local buildings (less than 1 km from the flux tower, but outside the flux footprint). These measurements, paired with the large CO fluxes observed in the winter evenings, suggest that there is the potential for advection contamination affecting our measurements at the field site.

**Fig. 5:**
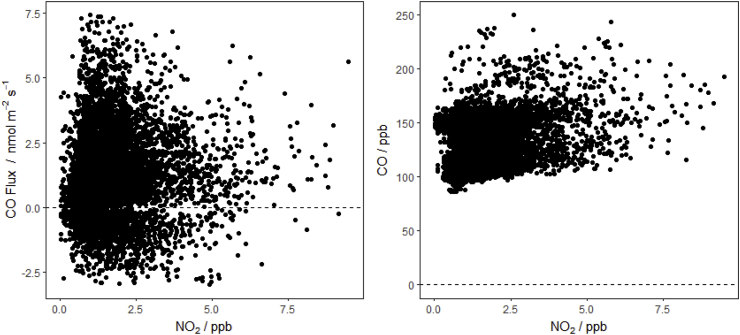
Measured CO Flux and CO concentration plotted against NO_2_ concentrations recorded at the field site between April 2016 and November 2017.

Due to the nature of the measurements (i.e. the direct comparison of flux with concentration and the high signal to uncertainty ratio of both datasets) it is not possible to clearly identify when horizontal advection from nearby combustion sources is affecting the measurements, or the magnitude that the individual half-hourly fluxes are affected. As the CO flux from the soils, litter and vegetation is often relatively small compared to the potentially large concentrations of CO released by nearby combustion, much of the potential contamination is indistinguishable when comparing CO and NOx measurements (Fig. 5). It is clear from the measurements that CO fluxes in the warmer months are almost entirely the result of meteorological conditions when NOx concentrations at the site are lowest, suggesting contamination is negligible during these periods.

### 3.3 Relationships between environmental factors and CO fluxes

The majority of flux measurements spanned a limited dynamic range and occurred when temperatures were mild (10–15 °C) and solar radiation was below 500 W m^−2^, therefore limiting the potential to investigate the separate effects of the correlated meteorological variables. Due to the complexity of the abiotic and microbiological processes all occurring in tandem, it is difficult to assess the total CO uptake and emission from any particular process at a given time. A total of 23% of CO flux measurements that passed quality control were negative. Although many of these individual negative fluxes are likely due to measurement uncertainty, on occasions negative fluxes were sufficiently consistent to provide strong evidence of real CO uptake (see April & May 2017 in Fig. 3). It is likely that even during peak emission times when net flux is highest (i.e. mid-day), that CO uptake continues to occur in the soil.

**Fig. 6:**
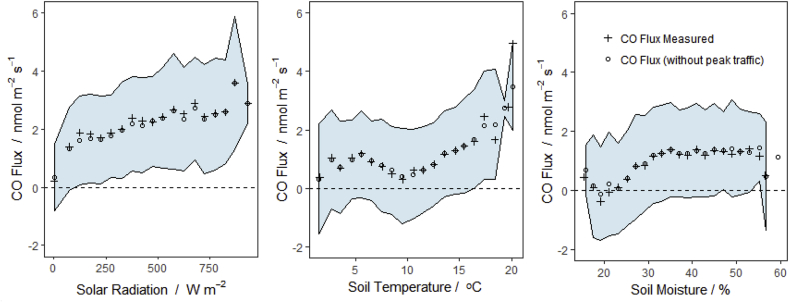
Mean of CO flux data binned by meteorological conditions during measurement (with standard deviation as shaded range). Data without fluxes measured during traffic peak times (6–9 a.m. and 4–8 p.m.) are included for comparison (dots).

Statistically binning the flux data across the total spectrum of environmental conditions reveals the extent to which fluxes vary accordingly (Fig. 6). In this assessment it becomes clear that there is a tendency for fluxes to increase with solar radiation, but the relationship with soil temperature and soil moisture is less consistent. While the photo-degradation of hydrocarbons is entirely an abiotic process, microbial activity can also be associated with temperature.

## 4 Discussion

### 4.1 CO fluxes from managed grassland

In this study we present the first long term (longer than 12 months) eddy covariance dataset of CO fluxes measured from a grassland source in the UK. Until now, very few long-term studies have been published globally regarding CO fluxes from semi-natural sources, especially using the eddy covariance method. Due to instrumentation and logistical constraints, much of the previous work carried out in the field of CO has been limited to smaller studies with few data points. This study shows by using recently developed, commercially available instrumentation, that long-term, undisruptive measurements of CO flux at the field scale are now feasible for future studies.

Our study reveals that CO fluxes from the grassland varied between positive and negative dependent upon the meteorological conditions. Emissions followed a consistent diurnal cycle of high and low fluxes, occasionally becoming negative. Negative fluxes were most visible during the night, especially when soils were at their driest. It is uncertain from our measurements if the response to drier soils is due to favourable conditions for microbial activity or if it is a physical response leading to increased interaction between soil surface area and the atmosphere due to more porous soil. The fluxes reported in this study are similar in magnitude to those observed in other local studies using chamber measurements which report CO fluxes between −1 and 14 nmol m^−2^ s^−1^ (median of 1.92 nmol m^−2^ s^−1^) (Moxley and Cape, 1996; Moxley and Smith, 1997).

The diurnal patterns and negative fluxes of CO have been described in detail for bioenergy crops (Pihlatie et al., 2016), as well as agricultural and forest soils (King, 2000; Asperen et al., 2015). It is consistently reported that CO fluxes correlate well with solar radiation and/or soil temperature in these studies. Using the GAM method we are able to predict CO flux using only solar radiation, soil temperature and soil moisture measurements with an R^2^ of 0.37 on a 30 min basis. Although limited by relatively high random uncertainty in CO flux measurements, this method shows that much of the diurnal variation in observed fluxes at the site can be explained by these three variables. This does not come as too much of a surprise as we would expect photo and thermal degradation as well as microbial processes to rely largely on these environmental factors, but the exact processes at work cannot be explained any further in this study with the available data.

Further comparisons with the individual meteorological drivers reveal an apparent dip in CO emissions at approximately 10 °C with a soil volumetric moisture content at approximately 20%. These conditions may indicate favorable conditions for microbial uptake of CO in soils as any abiotic relationships between flux and environmental conditions would be expected to follow a more predictable trend: however, there is likely some interaction between the three variables and further in-depth research would be required to verify the consistency of these findings.

### 4.2 Annual carbon budget

**Fig. 7:**
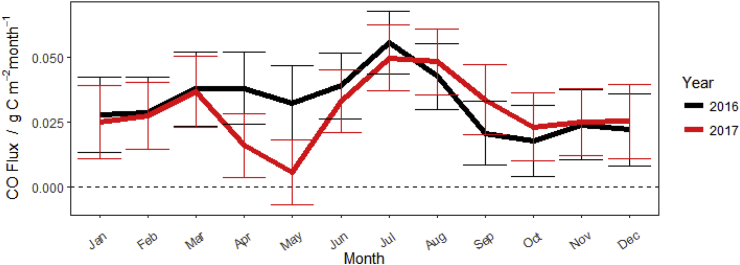
Monthly fluxes of CO measured and gap-filled for 2016 and 2017 (with 95% confidence intervals).

The GAM was used to gap fill the measured CO fluxes to provide annual cumulative flux values for both 2016 and 2017, replacing missing flux values with the prediction based on the fitting with solar radiation, soil temperature and soil moisture at a 30 min interval. The GAM fit is used solely to estimate net fluxes and does not provide a meaningful summary of the significance of the model fit for each of the individual inputs; however, the impact of seasonal weather conditions on emissions are apparent over the measurement period. A weak seasonal trend is observed in the data with fluxes highest during the warm sunny months of summer (Fig. 7). Soil moisture content appears to have a major impact on emissions, distorting the expected seasonal trend (as is observed in the dry Spring, 2017).

Annual fluxes (and 95% confidence intervals) were estimated as 0.38 ± 0.046 and 0.35 ± 0.045 g C m^−2^ y^−1^ for 2016 and 2017, respectively. Annual CO flux estimates based on measurements are rare and vary between different ecosystems. Previous estimates made from measurements taken from bioenergy crops and forest soils vary from 0.03 g C m^−2^ y^−1^ to −0.11 g C m^−2^ y^−1^(Constant et al., 2008; Pihlatie et al., 2016; Sun et al., 2018). Globally the net uptake and release of CO is expected to vary in different climatic zones, but generally soils are considered a net sink of CO (Liu et al., 2018) which is not reflected in our study. As our study focused on the net emission from the grassland, we were not able to determine if CO emissions were from organic materials in the soil, or from plant litter present on the surface. Further research would be necessary to quantify the contributions of each of these materials separately. The comparison of CO fluxes reported in this study with the reported net ecosystem exchange (NEE) in the field (Jones et al., 2017) shows that carbon losses in the form of CO are insignificant (0.17%) when compared to the measured uptake of CO_2_ in the field (218 g C m^−2^ y^−1^).

Total emissions of CO from the UK were estimated at 1614 Gg in 2015 (Brown et al., 2017), following a downward trend from values above 7000 Gg in the early nineties. These estimates are based almost entirely on fuel combustion. Considering that the UK land area coverage is predominantly arable and grassland (approximately 70%) (CEH Land Cover Map, 2015), if the CO fluxes reported in this study are representative of these areas then national annual emissions could be expected to be in the order of 61.91 (54.3–69.5) Gg, which equates to 3.8% (3.4–4.3%) of the current inventory total. Although measurements are limited to one site in this study and meteorological conditions will vary significantly at a national scale, this potential contribution is significant enough to merit further study and consideration in comparison to annual UK CO inventories. Further investigation is definitely required to better understand CO fluxes from terrestrial areas at a national scale in order to better represent the variety of sources.

### 4.3 Further considerations

Individual flux measurements of CO were small with a relatively high degree of uncertainty. The GAM used in this study was able to fit well with the large 20 month long dataset due to the long-term measurement data available and high quality of the gas analyser. Our study suggests that short term experiments and using less developed instrumentation and methodology may struggle to provide the data essential to capture the variability of CO fluxes across the seasons. Additionally, had NOx measurements not been available at the site, we would not have been able to identify elements of contamination from combustion in our measurements, nor identify when contamination was affecting measurements (i.e. winter). The GAM used in this study to fit flux data with meteorological variables is largely unaffected by the contamination at the site as the vast majority of data that passed quality control was either recorded during the warmer months or out with rush hour timing (R^2^ ≈ 1 between GAM predictions fitted with and without rush hour traffic times); however, this was more by good fortune than planning. We suggest that present and future work which intends to monitor CO emissions from terrestrial sources should include atmospheric NOx concentration/flux measurements at the same frequency to ensure that any contamination can be identified.

## 5 Conclusions

This study shows that the intensively grazed grassland field site was a net source of CO, emitting an estimated 0.37 ± 0.045 g C m^−2^ y^−1^. Fluxes from the field followed a consistent diurnal cycle, peaking at midday and returning to values near zero at night. Fluxes also varied between positive and negative depending on the meteorological conditions, primarily solar radiation, soil temperature and soil moisture content. Our measurements revealed that uptake of CO was most likely to happen at night, at temperatures near 10 °C in soils with volumetric water content between 10 and 20%. The overall carbon losses from the field can be considered negligible at the site, accounting for only 0.17% of the total NEE associated with CO_2_ flux.

Our results highlight that although terrestrial sources of CO are relatively small, at a national scale these emissions should not be ignored in chemistry and transport models, potentially accounting for up to 4.3% of the UK inventory total. We recommend that further work is carried out to investigate CO fluxes from other terrestrial sources such as carbon rich peat bogs and even synthetic hydrocarbon surfaces exposed to sunlight (i.e. tar and bitumen) in order to further understand and mitigate the problematic effects of elevated atmospheric CO.
